# Influence of Mg, Cu, and Ni Dopants on Amorphous TiO_2_ Thin Films Photocatalytic Activity

**DOI:** 10.3390/ma13040886

**Published:** 2020-02-17

**Authors:** Vytautas Kavaliunas, Edvinas Krugly, Mantas Sriubas, Hidenori Mimura, Giedrius Laukaitis, Yoshinori Hatanaka

**Affiliations:** 1 Department of Physics: Faculty of Mathematics and Natural Sciences, Kaunas University of Technology, Studentų str. 50, 51368 Kaunas, Lithuania; mantas.sriubas@ktu.lt (M.S.); giedrius.laukaitis@ktu.lt (G.L.); 2 Graduate School of Science and Technology, Shizuoka University, 3-5-1 Johoku, Naka-Ku, Hamamatsu, Shizuoka 432-8011, Japan; 3 Faculty of Chemical Technology, Kaunas University of Technology, Radvilėnų pl. 19, 50299 Kaunas, Lithuania; edvinas.krugly@ktu.lt; 4 Research Institute of Electronics, Shizuoka University, 3-5-1 Johoku, Naka-Ku, Hamamatsu, Shizuoka 432-8011, Japan; mimura.hidenori@shizuoka.ac.jp (H.M.); nrd06083@nifty.com (Y.H.)

**Keywords:** amorphous materials, titanium dioxide thin films, sputtering, catalytic properties, photocatalysis

## Abstract

The present study investigates Mg (0 ÷ 17.5 wt %), Cu (0 ÷ 21 wt %) and Ni (0 ÷ 20.2 wt %) dopants (M-doped) influence on photocatalytic activity of amorphous TiO_2_ thin films. Magnetron sputtering was used for the deposition of M-doped TiO_2_ thin films. According to SEM/EDS surface analysis, the magnetron sputtering technique allows making M-doped TiO_2_ thin films with high uniformity and high dopant dispersion. Photocatalysis efficiency analysis was set in oxalic acid under UV irradiation. In accordance with the TOC (total organic carbon) measurements followed by the apparent rate constant (*k_app_*) results, the dopants’ concentration peak value was dopant-dependent; for Mg/TiO_2_, it is 0.9% (*k_app_*—0.01866 cm^−1^), for Cu/TiO_2_, it is 0.6% (*k_app_*—0.02221 cm^−1^), and for Ni/TiO_2_, it is 0.5% (*k_app_*—0.01317 cm^−1^). The obtained results clearly state that a concentration of dopants in TiO_2_ between 0.1% and 0.9% results in optimal photocatalytic activity.

## 1. Introduction

Photocatalysis and photocatalytic materials based on semiconductors have been studied for more than a decade [1,2,3]. By reason of higher efficiency and future potential in water treatment [4], air purification [5] and even hydrogen production [6], photocatalysis interest increased significantly. The number of articles related to photocatalysis has risen thousands of times over the past 20 years. Additionally, research based on TiO_2_ as a photocatalyst among other semiconductors also increased [7]. TiO_2_ manufacturing is affordable, which makes it economically favorable, especially when compared with materials having similar properties, such as SnO_2_, CeO_2_, CdS, and WO_3_. These semiconductors are also used for photocatalysis research because of their biocompatibility, stability in various conditions, and capability to generate excitons [8,9]. TiO_2_ achieves better photocatalytic activity than ZnO [10] or CdS [11] and under the same conditions; not only does it have better photochemical stability, but TiO_2_ is superior photocatalyst compared to WO_3_ [12]. TiO_2_ has great potential in energy and environmental research, such as uses in lithium-ion batteries, self-cleaning coatings, water purification, or as a catalyst for photocatalytic reactions [13,14,15,16]. As a result of its unique dielectric and optical properties, TiO_2_ is considered as a non-hazardous material and could be modified, depending on the requirements for specific applications [17]. It is known that the TiO_2_ band gap is approximately 3–3.2 eV (depending on phase) and its conduction band minimum (CBM) is almost as same as hydrogen potential, while the valence band maximum (VBM) is slightly lower than oxygen potential, at approximately 1.6 eV [18,19,20].

The efficiency and photocatalysis properties depend on the crystal structure of TiO_2_. TiO_2_ has three common phases: rutile, anatase, and brookite (least studied because of its instability). Rutile is the most thermodynamically stable phase, as demonstrated when anatase and brookite slowly change phases to rutile at 550–750 °C [21]. Both anatase and rutile have a tetragonal structure, which can be distinguished from one another because of their crystal habit. Anatase differs from rutile in that its octahedrons share four crystal edges, forming a four-fold axis. The TiO_2_ anatase phase particle surface possesses a triangular arrangement, which creates a reaction condition with the adsorbed molecules and achieves a slightly higher reduction rate and absorption of organic molecules. Studies show that the photocatalysis process is more efficient with rutile and anatase phases than brookite [22,23,24], and it is best with a mixture of anatase and rutile (70% and 30% respectively, called Degussa P25) [25,26,27]. Even though the chemical structure of the anatase or rutile phase seems to be more favorable for photocatalysis, it is still inefficient in its pure phase [26,28,29]. Nevertheless, based on research and interest, amorphous TiO_2_ stands alongside crystalline TiO_2_ as an alternative to crystalline structures [30,31,32]. According to Kaur et al. (2012), the electronic structure of amorphous TiO_2_ is similar to that of crystalline TiO_2_ but with a larger band gap [33]. Nonetheless, the energy band gap can be controlled with dopants. The study of Khramov et al. shows that the modification of Degussa P25 with other metals can lead to the amorphization of TiO_2_ structure [34]. Based on that, it appears that not only do the dopants change the electrical structure of TiO_2_ (by modifying the band gap) and increasing the photocatalytic efficiency, but this could also lead to changes in the TiO_2_ phase structure (amorphization), which would therefore decrease the photocatalytic efficiency because an amorphous structure has a higher number of recombination centers. According to Shu et al., the amorphous TiO_2_ films have many defects, which increases their conductivity. However, those defects appear mostly inside the film, which leaves TiO_2_ as a semiconductor with an energy band gap nearly to the dielectric. Despite that, doping the TiO_2_ with another metal can increase the surface conductivity, which allows easily transfering charge carriers [35]. This constructs the potential of amorphous TiO_2_ as a low-cost competitor to crystalline phase TiO_2_. Controversially, another study declares that amorphous TiO_2_ has negligible photocatalytic activity, due to its defective states [36]. Over the last two decades, studies of crystalline and amorphous TiO_2_ show debatable results when comparing the photocatalysis efficiency based on the TiO_2_ structure [31,37], which leaves this field of study open for further research.

The electrical properties are the main parameters for the semiconductor in the photocatalysis process. The efficiency of the process can be influenced by particle size, phase composition, crystal structure, or even purity of the samples [38,39]. Electrical properties can vary depending on the deposition technique and parameters [40,41,42]. Moreover, the photocatalytic properties can be modified by adding dopants to the film or on the surface [43,44], and/or by changing the surface/bulk structure of TiO_2_. Thus, metal ions act as electron trappers, generators of excitons, and/or as photosensitizers, raising light absorbance under visible light. Transition metal ions change the conduction band minimum (CBM) or valence band maximum (VBM) energy levels, which reduce the energy band gap or create additional levels in reducing exciton recombination time [45,46,47]. Furthermore, the electron work function of metal dopants must be considered beforehand. Metal dopants with an electron work function (*Φ_m_*) similar to the amorphous TiO_2_ Fermi energy level (*E_F_*) could enhance the photocatalysis process significantly, and vice versa.

The aim of this research is to find an optimal concentration of Cu, Ni, and Mg dopants in amorphous TiO_2_ thin films, and to investigate the photocatalytic activity of the formed thin-film structures. Thin-film formation and analysis were done under the same conditions for a better comparison. TiO_2_ thin films with different concentrations of Mg, Cu, and Ni dopants were formed using the magnetron sputtering deposition method, and photocatalytic activity was tested using an oxalic acid solution.

## 2. Experimental Method

### 2.1. Films Preparation

**Figure 1:**
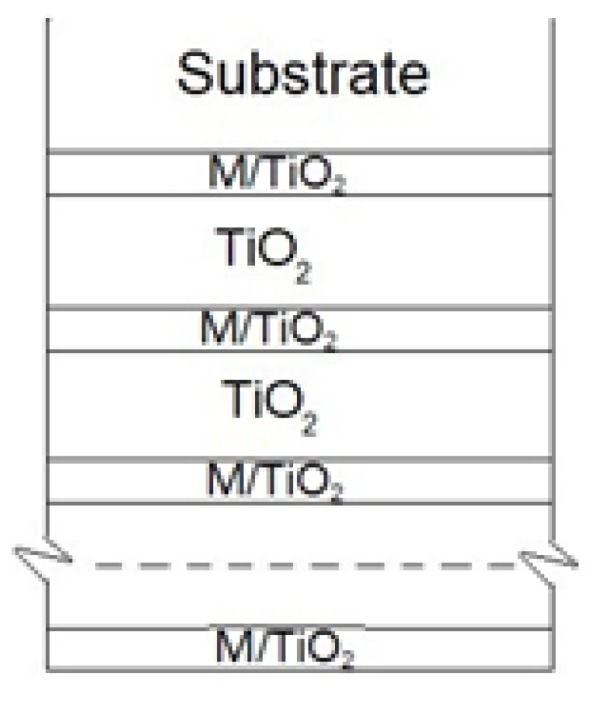
M-doped TiO_2_ thin film structure.

Thin films were deposited using the Kurt J. Lesker PVD 75 vacuum system with four magnetron sputtering stages. Argon and oxygen gases (with 99.999% purity) were used during the plasma activation and deposition processes. Two titanium (Ti) targets (99.995% purity), a copper (Cu) target (99.99% purity), a magnesium (Mg) target (99.95% purity), and a nickel (Ni) target (99.995% purity), all purchased from Sigma Aldrich^®^, were used for thin-film formation. Power sources including direct current (DC), pulsed DC, and radio frequency (RF) were used. The DC source was used for the Ti and Ni targets, pulsed DC was used for the Mg target, and RF was used for the Cu target. TiO_2_ and Cu, Ni, and Mg-doped TiO_2_ films were deposited on stainless steel plates (304 series). The substrate was cleaned in pure acetone (for 10 min) using an ultrasonic bath in order to avoid any organic contamination. Thin films were deposited at 300 °C maintaining the ratio of oxygen and argon at 20/80. M-doped TiO_2_ were produced using two titanium cathodes (DC source) and one metal cathode (DC, pulsed DC, or RF source) target. Different output power and shutter open/close ratios were used to achieve different concentrations of M in TiO_2_ films. The total thickness of the formed thin films was around 100 nm; the structure based on the deposition procedure is shown in Figure 1. Different Cu, Ni, and Mg concentrations in TiO_2_ thin films were achieved (Table 1), i.e., Mg, 0 ÷ 17.5 wt %; Cu, 0 ÷ 21 wt %; and Ni, 0 ÷ 20.2 wt %. The concentration of dopants in TiO_2_ was analyzed via X-ray photoelectron spectrometer (XPS) and energy-dispersive X-ray spectroscopy (EDS).

### 2.2. The Morphology and Structural Analysis

The structure of the TiO_2_ phase was determined using an X-ray diffractometer (XRD) “Bruker D8 Discover” at 2Θ angle in a 20° to 70° range using Cu Kα (λ = 0.154059 nm) radiation, 0.01° step, and a Lynx eye position-sensitive detector (PSD).

The surface topography images were obtained via scanning electron microscope (FE-SEM; JEOL, SM-71010) using 8 kV accelerating voltage.

The distribution of elements (mapping) and a high concentration of dopants were measured using an energy-dispersive X-ray spectroscope “BrukerXFlash Quad 5040” (EDS) with an accelerating voltage of 10 kV.

The low concentration of dopants in TiO_2_ was determined by the X-ray photoelectron spectrometer (XPS, PHI Versaprobe 5000). Measurements were performed using monochromatic X-ray radiation (Al Kα, 1486.6 eV), 25 W power, 100 μm beam size, and the measurement angle was 45° during the experiments. The sample charging effect was compensated using the radiation of low energy electrons and ions. The resolution was 1 eV for Survey Spectrum and 0.1 eV for detailed chemical analysis. The pass energy was 187.580 eV. Thin films were sputtered in order to achieve the depth profile of the samples [48].

### 2.3. Photocatalysis Efficiency Evaluation

**Figure 2:**
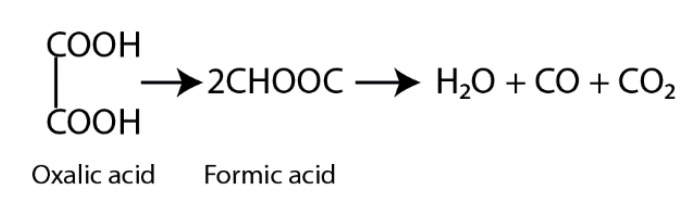
Dehydration of oxalic acid molecule.

To maximize the efficiency of the photocatalysis process, a transparent solution must be chosen. There are many types of research where methylene blue (MB) [49,50], rhodamine B (RhB), [51,52] or other organic dyes were selected as a solution for this process. In that case, the light absorption of the solution itself and the degradation process happening without the photocatalyst should be considered as an extraneous effect, which could cause slightly inaccurate results of the semiconductor as a photocatalyst [53,54,55]. For this reason, measurements were set in oxalic acid solution (which is as well a product of RhB during the photodegradation process) made from water and oxalic acid powder (with an initial concentration of 100 mg/l) under UV light (light peak at 254 nm, 18 W power). According to the light source used in the experiment, absorption spectra were measured but not analyzed in this manuscript because of the same absorbance at 254 nm for all the samples. In this range of light, metal dopants do not have an effect on the absorption characteristics of TiO_2_. The samples (surface area—1600 mm^2^) were immersed in the solution and set for 30 min in the dark before every experiment. After that, the samples were irradiated for 80 min, and the solution was continuously stirred using a magnetic stirrer. The distance between the UV lamp and the sample was set to 50 mm, while the distance between the surface of the solution and the sample was 20 mm. Samples of solution were taken after 20, 40, 60, and 80 min of irradiation, and the degradation of the oxalic acid solution was measured in a Shimadzu TOC-L (Shimadzu Corp. Japan) analyzer according to the EN 1484:2002 procedure. To minimize errors, there were three measurements for each sample taken from the solution, and the average concentration was analyzed. The degradation steps of oxalic acid solution in water are shown in Figure 2. Initially, C_2_H_2_O_4_ decomposes into formic acid, and with further degradation into the innocuous compounds (H_2_O and CO_2_), through intramolecular dehydration.

The results were evaluated based on the thermodynamics and kinetics of the process. The driving force of photocatalysis is an organic solution and oxygen molecules. Oxygen is constant in photocatalysis because the experiment is set in the atmospheric ambient and the solution is surrounded by oxygen molecules. The adsorption of organic compounds on the surface of the photocatalyst is the kinetic driving force of the photocatalysis process. Adsorption and desorption equilibrium follows Langmuir isotherm [56,57]. In this case, under UV irradiation, there is an equilibrium in adsorbed and desorbed molecules.

This evaluation is useful when adsorption and desorption are in equilibrium [58], but because the photocatalysis efficiency is low, it could be applied as a universal equation for different solutions:

$$k_{app}=\frac{1}{t}\ln\left(\frac{C_{0}}{C_{t}}\right),$$

where *k_app_*—apparent rate constant, *C_O_*—initial concentration, *C_t_*—concentration in time, and *t*—time. Keeping in mind that *k_app_* depends on the time and concentration differences in time, average meanings were calculated for the different concentrations of dopants in TiO_2_ (Table 1).

## 3. Results and Discussion

**Figure 3:**
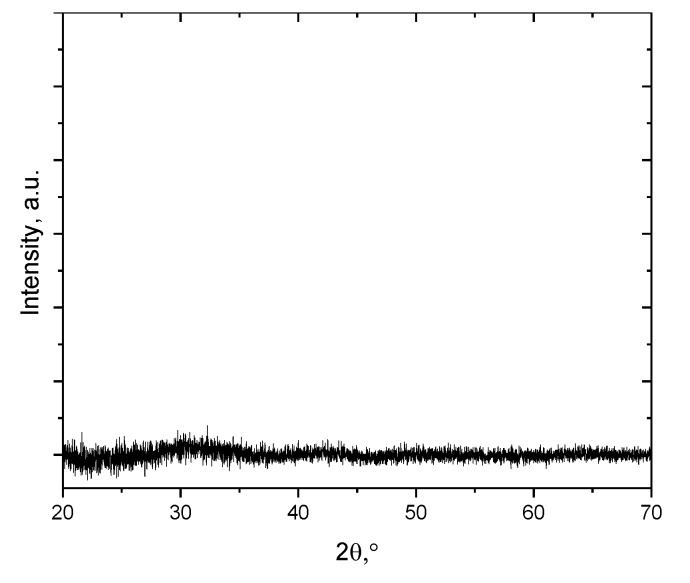
XRD pattern spectra of amorphous TiO_2_ without dopants.

The main parameters for a dopant to be effective as a charge trapper are its concentration and dispersion in TiO_2_ lattice, as well as its electron configuration. X-ray Diffraction (XRD) analysis shows that deposited TiO_2_ thin films were amorphous (Figure 3).

**Figure 4:**
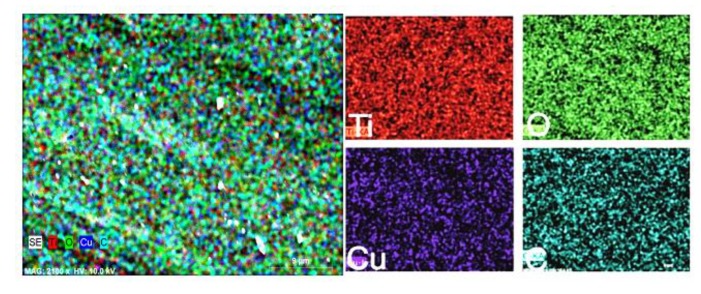
Cu (0.6 wt %) doped TiO_2_ sample surface elemental analysis.

According to elemental analysis (Figure 4), there is a high dispersity of dopant clusters in TiO_2_ thin films. Cluster dispersity defines photocatalysis efficiency, since dopant clusters act as electron trappers. Based on the results (Table 1) and theory, cluster size and dispersity, as well as photocatalysis efficiency, depend on dopant concentration. Thus, theoretically, the same efficiency could also have a similar dependence on size and dispersity.

**Figure 5:**
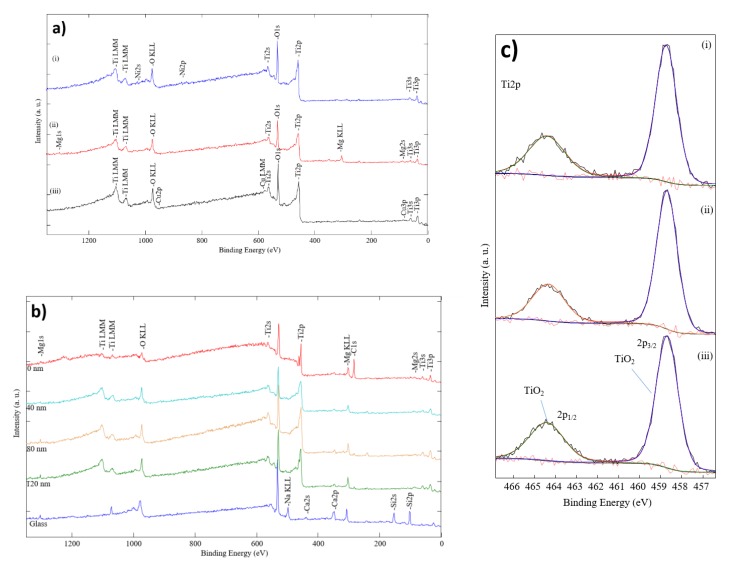
XPS spectra of formed M-doped TiO_2_ thin films: (**a**) survey; (**b**) depth profiling of Mg-doped TiO_2_ thin films; and (**c**) Ti2p; dopant (i) Ni, (ii) Mg and (iii) Cu.

Additionally, XPS analysis was set for samples with a low concentration of dopant in TiO_2_. The calibration of XPS spectra was done according to the standard value of C 1s peak at 284.8 eV BE (binding energy). The reference data for XPS peaks were taken from the “Thermo Fisher” (Thermo Fisher Scientific Inc.) database. After the calibration, core level peaks of TiO_2_ (Figure 5c) and dopants (Figure 5a) were measured. Ti 2p_3/2_ and Ti 2p_1/2_ peaks are at 458.5 eV and 464.5 eV BE (compared to TiO_2_ Ti 2p_3/2_—458.5 eV BE and Ti 2p_1/2_—464.5 eV BE). The principal Mg KLL Auger peak is at 305 eV BE, accompanied with an Mg 1s peak at 1303 eV BE (ref. Mg 1s—1303 eV BE for Mg metal and 1304.5 eV BE for Mg native oxide). Furthermore, it is hard to distinguish Cu/TiO_2_ spectra if a Cu_2_O or Cu structure formed because of low concentration and low peak intensity. Cu 2p_1/2_ and Cu 2p_3/2_ peaks are at the same energy value of 933 eV BE and 955 eV BE (accordingly). There is also a weak satellite at 945 eV BE, which helps to separate Cu metal and Cu_2_O. The Ni 2p_3/2_ peak is at 855 eV BE (ref. Ni metal—852.6 eV BE; NiO at 853.7 eV BE). The depth profile of doped TiO_2_ thin films is shown in Figure 5b. In agreement with XPS results, dopants clusters are not oxidized, while the main dopant metal peaks are at the same energy value according to the reference. However, based on the deposition parameters, where an oxygen and argon ratio at 20/80 was used, we consider that at some points, the dopant material could be slightly oxidized.

The evaluation results (Table 1) show that increasing the concentration of dopant above 1 wt % in TiO_2_ thin films results in a decrease in the efficiency of the photocatalysis process. Although, if the concentration of dopant is lower than 1 wt %, the efficiency increases in the first 20 min of irradiation, while the optimal amount of concentration depends on the dopant itself. In this study, Cu-doped TiO_2_ reaches maximum efficiency at 0.6 wt %, Ni-doped TiO_2_ reaches maximum efficiency at 0.5 wt %, and Mg-doped TiO_2_ reaches maximum efficiency at 0.9 wt %. Therefore, the optimal concentration value suggests that there is an optimal cluster size for maximum efficiency.^42^ Efficiency dependence on cluster dispersity and size has not been investigated in this study.

**Figure 6:**
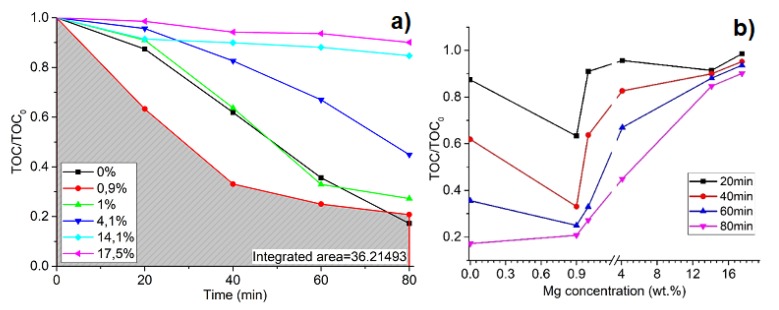
Degradation of oxalic acid in time as a function of Mg concentration in TiO_2_ thin films, where (**a**) degradation of oxalic acid in time at different Mg wt % concentrations and (**b**) photocatalysis process dependence on Mg wt % dopant concentration.

The degradation of oxalic acid in time at different Mg wt % concentrations is shown in Figure 6a. The integrated area under the curve (gray area) indicates the photocatalysis process efficiency as well as apparent rate constant *k_app_*. The smaller the area and the higher the constant, the higher the process efficiency. The calculated values show that the higher efficiency of Mg/TiO_2_ as a photocatalyst is achieved when the dopant concentration in the TiO_2_ sample is 0.9 wt % (*k_app_* = 0.01866 min^−1^). The photocatalysis process dependence on dopant concentration is shown in Figure 6b. Compared to pure TiO_2_ thin films, *k_app_* drops significantly when the concentration of Mg is increased by more than 1 wt %. There is a peak in concentration where the photocatalysis efficiency reaches maximum values. It could be stated that there should be a peak with a lower concentration of Mg. As Manzanare’s research shows, a small amount of Mg dopant in the TiO_2_ lattice increases the electron trap (Ti^3+^) and hole trap (O_S_) concentration, providing a low recombination of electron–hole pairs [59]. On the other hand, Mg clusters on the TiO_2_ surface can act as an electron donor for TiO_2_. In this way, oxygen can be directly affected by injected electrons and followed by easily formed oxygen vacancies on the unstable TiO_2_ surface [60]. Different concentrations of Mg were used; the most efficient concentration was 0.9 wt %, while 1 wt % was lower but still higher than pure TiO_2_.

**Figure 7:**
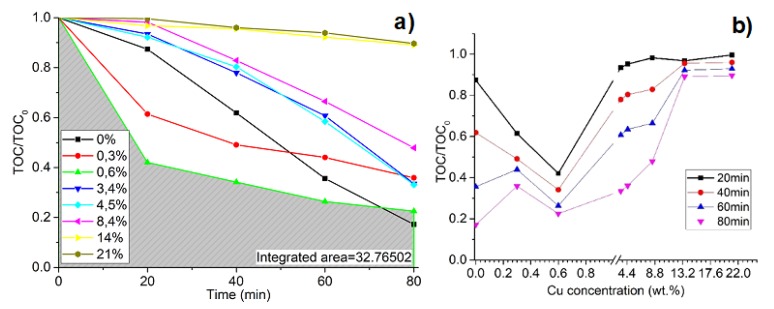
Degradation of oxalic acid in time as a function of Cu concentration in TiO_2_ thin films, where (**a**) the degradation of oxalic acid in time at different Cu wt % concentrations and (**b**) the photocatalysis process dependence on Cu wt % dopant concentration.

Accordingly, the same measurements were set with Cu and Ni dopants as well. Depending on the integrated area under the curve (Figure 7a), Cu (0.6 wt %) is more efficient as dopant compared to Mg (0.9 wt %). Furthermore, comparing the same samples, after the first 20 min, 58% of solution degraded with Cu/TiO_2_ and only 37% degraded with Mg/TiO_2_ (Figure 6b and Figure 7b). Cu concentration values between 0.5 and 0.7 are considered optimal concentrations where the degradation of oxalic acid increases drastically. Decreasing or increasing the Cu concentration in the TiO_2_ lattice negatively affects the efficiency of the process. The sample with 0.6 wt % of Cu reaches the maximum efficiency (*k_app_* = 0.02221 min^−1^), while pure TiO_2_ reaches the maximum efficiency at *k_app_* = 0.01160 min^−1^, and the sample with 0.3 wt % of Cu reaches the maximum efficiency at *k_app_* = 0.01373 (Table 1). Bensouici’s studies show that the pure TiO_2_ reaction rate constant is *k_app_* = 0.015 min^−1^, and it decreases to *k_app_* = 0.001 min^−1^ for Cu 4 wt % and remains stable for 6–10 wt % Cu-doped TiO_2_ thin films [61]. In this study, there was a quite huge change in concentration between 0.3 wt % and 2 wt %, while in Minsu Jung’s research [62], a concentration of 0.5 wt % Cu in the TiO_2_ lattice reaches the highest H_2_ production rate, and decreasing or increasing this value lowers the production of H_2_. However, other research states that Cu is not an effective dopant for TiO_2_ modification. With the increased concentration of Cu in TiO_2_, the yield of a product decreases, as compared to pure TiO_2_ [63]. However, analyzing the dependency of dopant concentration on photocatalysis efficiency, low concentrations must be tested by slowly increasing them. According to this statement, Tashibi et al. analyzed 0.2 wt % and 0.9 wt % of Cu in TiO_2_, skipping the crucial point of approximately 0.5 wt % where photocatalysis efficiency reaches higher values when compared to pure TiO_2_. Despite that, this research explains the inactivity of Cu-doped TiO_2_ by the formed CuO on the surface, which decreases the number of active sites of TiO_2_ as well as becomes a recombination center and increases the rate of the charge carrier recombination. However, the optimal amount of clusters results in the dispersion of them enough to reduce recombination rates and act as charge trappers. The photodegradation of oxalic acid reaches its maximum efficiency when the Cu concentration is 0.6 wt % in TiO_2_ films, and similar to other studies, the efficiency drops when the concentration is raised or lowered. Different thin film deposition techniques, preparation, and materials (solid, liquid, or gas phase) can cause slightly different results in the experiment. Thin-film parameters such as the crystal size, purity, and surface area apart from the crystal phase rely on deposition techniques, as described earlier in this article. However, a different evaluation method can cause varied results in photocatalysis efficiency.

**Figure 8:**
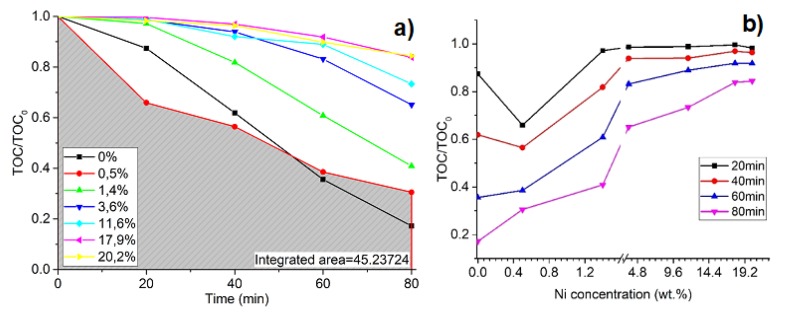
Degradation of oxalic acid in time as a function of Ni concentration in TiO_2_ thin films, where (**a**) degradation of oxalic acid in time at different Ni wt % concentrations and (**b**) photocatalysis process dependence on Ni wt % dopant concentration.

Ni/TiO_2_ thin films as photocatalyst results analysis show that the photodegradation of oxalic acid is efficient with 0.5 wt % of Ni in the TiO_2_ lattice (Figure 8a), but it is similar to pure TiO_2_ thin films results. After 20 min, only 34% of oxalic acid solution degrades, which is similar to 0.9 wt % Mg, but taking results after 40 min in comparison, for Mg, it is 67% (Figure 6b), and for Ni, it is 43% (Figure 8b). Chen’s [64] studies show that the Ni/TiO_2_ photocatalyst exhibits the highest photocatalytic activity when the optimal Ni concentration is 0.5 wt %. Experimental results, while using oxalic acid as a solution for the examination of photocatalytic properties, and Ni/TiO_2_ as the photocatalytic show the same results. Keeping in mind that the degradation process depends on the adsorption of the solution on the surface, increasing the concentration of Ni on TiO_2_ can result in a decrease in surface irrigation. Other research based on Ni concentration in crystal phase TiO_2_ shows that there is an optimal concentration of Ni on crystal phase TiO_2_ as well, with which the photocatalytic efficiency increases drastically. The decrease in efficiency based on the concentration is explained by decreasing the number of active sites of TiO_2_ on the surface with increased Ni concentration [65]. With the right amount of Ni clusters on the TiO_2_ surface, Ni acts as a co-catalyst by separating and transferring photogenerated charge carriers, thus decreasing the recombination rates compared to those of pure TiO_2_ [66].

Based on the results in Figure 6, Figure 7 and Figure 8a, the degradation rate of the oxalic acid solution, while using optimal dopant concentration, drops exponentially. In the first 20–40 min, the degradation rate is higher compared to degradation after 40 min, when the reaction almost stabilizes. After the experiment with Cu (0.3 and 0.6 wt %), Mg (0.9 wt %) and Ni (0.5 wt %) doped TiO_2_ films, visible surface area degradation was observed. The thin film degradation was not detected using TiO_2_ with higher dopant concentration. The reactivation of such catalysts has to be taken into account considering the economic implications [67]. The strength of TiO_2_ films and adherence are relevant for repetitive applications. Investigations on such parameters were done in previous reports [68,69,70,71].

According to EDS mapping results (Figure 4), it can be stated that the PVD method is suitable for the formation of doped TiO_2_. Figure 4 shows the dispersion uniformity of Cu in TiO_2_, which means that active centers are dispersed evenly throughout the surface and in inside layers. The top M/TiO_2_ layer thickness (depending on the dopant) does not exceed 5 nm, and no annealing is done after deposition in order to keep amorphous TiO_2_.

**Figure 9:**
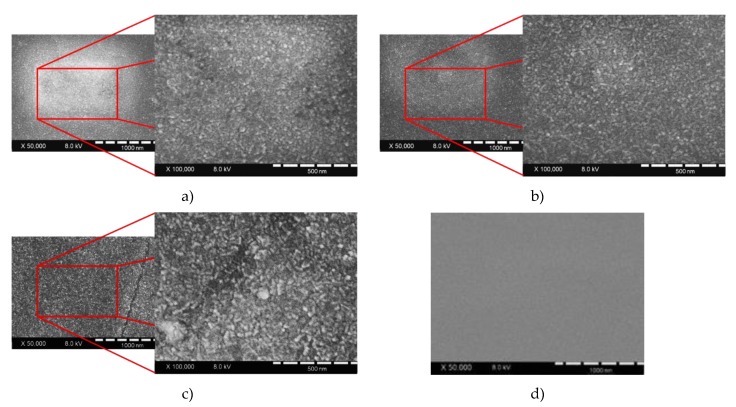
SEM pictures of (**a**) 0.9 wt % Mg/TiO_2_; (**b**) 0.5 wt % Ni/TiO_2_; (**c**) 0.6 wt % Cu/TiO_2_; (**d**) TiO_2_ thin films.

Since there are more charge trappers, a higher number of active centers lead to higher photocatalytic efficiency. Nevertheless, too many active centers can lead to small gaps between them and high recombination rates. It is known that the doping ratio depends on the particle size and cluster forming due to a high number of particles. On the other hand, having too many empty spaces leads to a lack of active centers, and recombination overcomes the charge-trapping process. Correlation between particle size and doping concentration is discussed by Jonathan et al. [72]. The SEM images show that the surface area of doped TiO_2_ has no defects, which emphasizes that high-quality films are formed via the PVD method. Moreover, according to SEM pictures, it can be noticed that the roughness of doped TiO_2_ is higher than that of pure TiO_2_ (Figure 9d). Moreover, the surface roughness of 0.6 wt % Cu/TiO_2_ (Figure 9c) is higher compared to others (Figure 9a,b), which can also lead to higher efficiency because of the increased surface area. The alignment between the electron work function of metal dopants and Fermi energy levels together with the CBM of TiO_2_ plays a big role in the photocatalysis mechanism.

**Figure 10:**
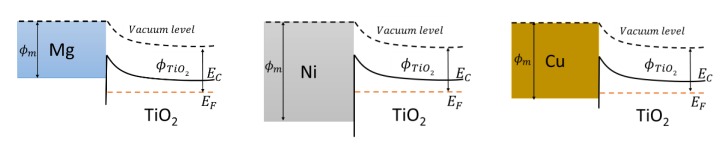
The alignment of the electron work function of metal dopants (*Φ_m_*) and titanium dioxide (*Φ*_TiO_2__).

The electron work function for dopants are *Φ_Mg_* = 3.66 eV; *Φ_Ni_* = 5.04 ÷ 5.35 eV; *Φ_Cu_* = 4.53 ÷ 5.10 eV; and the TiO_2_ electron work function is around 4.4 eV (*E_F_* − *E_C_* = 0.5 eV) [73]. Figure 10 shows a visual representation of the alignment of energy values. The differences in the electron work function work as a barrier and a certain amount of energy is required for an electron to overcome it. For Mg, it is 0.74 eV; for Ni, it is 0.64–0.82 eV; and for Cu, it is 0.13–0.5 eV. According to these results, the Mg dopant works as an electron donor, while Ni acts as an acceptor, and Cu could be a donor and acceptor at the same time, with Fermi energy almost aligned with the work function of Cu. These results coincide with previously stated results of the apparent rate constant, which showed that with a certain amount of Cu in TiO_2_, the photocatalytic activity was highest with the Cu dopant, followed by Mg and Ni [4].

## 4. Conclusions

The present research suggests that the magnetron sputtering technique is suitable for doped TiO_2_ film deposition because of its high purity, dopant dispersity in films, and the ability to deposit on a wide range of substrates. Mg-, Cu-, and Ni-doped TiO_2_ thin films were deposited as photocatalysts on alloy substrate, and the photocatalytic activity was determined by oxalic acid degradation under UV irradiation. Analysis revealed that based on the *k_app_*, with an increased dopant concentration (Mg 0.9 wt % → 4.1 wt %; Cu 0.6 wt % → 3.4 wt %; Ni 0.5 wt % → 3.6 wt %) process efficiency significantly drops accordingly (Mg *k_app_* 0.01866 min^−1^ → 0.00474 min^−1^; Cu *k_app_* 0.02221 min^−1^ → 0.00631 min^−1^; Ni *k_app_* 0.01317 min^−1^ → 0.00214 min^−1^). Equivalent results are with decreased dopant concentration: Cu 0.6 wt % → 0.3 wt %, efficiency drops *k_app_* 0.02221 min^−1^ → 0.01373 min^−1^. High photocatalysis efficiency is achieved with low exciton recombination rates. Since high concentration leads to small gaps between the active centers and the recombination process occurs, low concentration leads to a few active centers formed in the sample. Nevertheless, according to the TOC results, followed by the apparent rate constant, the Cu dopant achieved better photocatalysis efficiency compared to the Mg and Ni dopants. This can be explained by the formation of impurity energy levels in the TiO_2_ band gap.

## Figures and Tables

**Table 1:** Dopants concentration in samples and apparent rate constant k_app_ (where OA is an oxalic acid solution). Dopants concentration was analyzed via X-ray photoelectron spectrometer (XPS) and energy-dispersive X-ray spectroscopy (EDS).

Mg/TiO_2_		Cu/TiO_2_		Ni/TiO_2_		TiO_2_	OA
Conc., %	*k_app_*, min^−1^	Conc., %	*k_app_*, min^−1^	Conc., %	*k_app_*, min^−1^	*k_app_*, min^−1^	*k_app_*, min^−1^
0.9	0.01866	0.3	0.01373	0.5	0.01317	0.01160	0.00080
1	0.01015	0.6	0.02221	1.4	0.00519		
4.1	0.00474	3.4	0.00631	3.6	0.00214		
14.1	0.00227	4.5	0.00646	11.6	0.00170		
17.5	0.00092	8.4	0.00432	17.9	0.00152		
		13.5	0.00111	20.2	0.00114		
		21	0.00073				
